# Do changes in pulse pressure variation and inferior vena cava distensibility during passive leg raising and tidal volume challenge detect preload responsiveness in case of low tidal volume ventilation?

**DOI:** 10.1186/s13054-021-03515-7

**Published:** 2021-03-18

**Authors:** Temistocle Taccheri, Francesco Gavelli, Jean-Louis Teboul, Rui Shi, Xavier Monnet

**Affiliations:** AP-HP, Service de médecine intensive-réanimation, Hôpital de Bicêtre, DMU CORREVE, Inserm UMR S_999, FHU SEPSIS, Groupe de Recherche Clinique CARMAS, Université Paris-Saclay, 78, Rue du Général Leclerc, 94 270 Le Kremlin-Bicêtre, France

**Keywords:** Fluid responsiveness, Stroke volume variation, Acute respiratory distress syndrome, Fluid challenge

## Abstract

**Background:**

In patients ventilated with tidal volume (*V*t) < 8 mL/kg, pulse pressure variation (PPV) and, likely, the variation of distensibility of the inferior vena cava diameter (IVCDV) are unable to detect preload responsiveness. In this condition, passive leg raising (PLR) could be used, but it requires a measurement of cardiac output. The tidal volume (Vt) challenge (PPV changes induced by a 1-min increase in Vt from 6 to 8 mL/kg) is another alternative, but it requires an arterial line. We tested whether, in case of Vt = 6 mL/kg, the effects of PLR could be assessed through changes in PPV (ΔPPV_PLR_) or in IVCDV (ΔIVCDV_PLR_) rather than changes in cardiac output, and whether the effects of the Vt challenge could be assessed by changes in IVCDV (ΔIVCDV_Vt_) rather than changes in PPV (ΔPPV_Vt_).

**Methods:**

In 30 critically ill patients without spontaneous breathing and cardiac arrhythmias, ventilated with Vt = 6 mL/kg, we measured cardiac index (CI) (PiCCO2), IVCDV and PPV before/during a PLR test and before/during a Vt challenge. A PLR-induced increase in CI ≥ 10% defined preload responsiveness.

**Results:**

At baseline, IVCDV was not different between preload responders (*n* = 15) and non-responders. Compared to non-responders, PPV and IVCDV decreased more during PLR (by − 38 ± 16% and − 26 ± 28%, respectively) and increased more during the Vt challenge (by 64 ± 42% and 91 ± 72%, respectively) in responders. ∆PPV_PLR_, expressed either as absolute or as percent relative changes, detected preload responsiveness (area under the receiver operating curve, AUROC: 0.98 ± 0.02 for both). ∆IVCDV_PLR_ detected preload responsiveness only when expressed in absolute changes (AUROC: 0.76 ± 0.10), not in relative changes. ∆PPV_Vt_, expressed as absolute or percent relative changes, detected preload responsiveness (AUROC: 0.98 ± 0.02 and 0.94 ± 0.04, respectively). This was also the case for ∆IVCDV_Vt_, but the diagnostic threshold (1 point or 4%) was below the least significant change of IVCDV (9[3–18]%).

**Conclusions:**

During mechanical ventilation with Vt = 6 mL/kg, the effects of PLR can be assessed by changes in PPV. If IVCDV is used, it should be expressed in percent and not absolute changes. The effects of the Vt challenge can be assessed on PPV, but not on IVCDV, since the diagnostic threshold is too small compared to the reproducibility of this variable.

*Trial registration*: Agence Nationale de Sécurité du Médicament et des Produits de santé: ID-RCB: 2016-A00893-48.

**Supplementary Information:**

The online version contains supplementary material available at 10.1186/s13054-021-03515-7.

## Introduction

The oldest and most investigated way to predict fluid responsiveness during acute circulatory failure consists in measuring the respiratory variation in arterial pulse pressure (pulse pressure variation, PPV) and in stroke volume (stroke volume variations, SVV) during mechanical ventilation [1–4]. Nevertheless, PPV and SVV are limited because many clinical conditions affect their reliability. In particular, if tidal volume (Vt) is ≤ 8 mL/kg, many false negatives to these indices appear because the changes in cardiac loading conditions during ventilation are too small [3–5]. The respiratory variation of the diameter of the inferior vena cava (IVCDV) is also often used. Nevertheless, for the same reason as PPV and SVV, it should suffer from some false negatives in case of low Vt, even though this has been suggested by one study only [6] (see Additional file 1: Supplementary Table 1).

To work around this PPV limitation, some authors have suggested indexing it to changes in oesophageal pressure [7], which is not frequently measured, however. Two simpler methods have been proposed. The first is the passive leg raising (PLR) test. However, this test requires a direct measurement of cardiac output [8], which is not available in a significant number of patients. However, one can intuitively hypothesize that a decrease in PPV or IVCDV itself during a PLR test could indicate the presence of a preload dependence.

Another method for testing preload dependence in the event of low Vt is to perform a “Vt challenge” [9]. It consists in temporarily increasing the Vt from 6 to 8 mL/kg of predicted weight and observing the induced changes in PPV. A significant increase in PPV during a Vt challenge indicates the presence of a preload dependence [10] (Additional file 1: Supplementary Figure 1).

However, measuring PPV requires an arterial catheter or a non-invasive but expensive device [11]. An alternative to measure the effects of Vt challenge could be to measure its effects not on PPV, but on IVCV, the measurement of which only requires a transthoracic echocardiography.

Thus, the main aim of this study was to test whether the effects of a PLR test on PPV (ΔPPV_PLR_) and IVCV (ΔIVCDV_PLR_) reliably detect preload responsiveness when Vt is low. The secondary goals were to test whether the effects of a Vt challenge on IVCDV (ΔIVCDV_Vt_) can detect preload responsiveness and to confirm that IVCDV cannot do so in the case of Vt at 6 mL/kg.

## Patients and methods

### Patients

This prospective, interventional, one-centre study was carried out in the 25-bed medical intensive care unit of a university hospital. It has been approved by our institutional review board (Comité pour la protection des personnes Ile-de-France VII). All patients or their relatives gave informed consent.

The screening criteria were age ≥ 18 years, a transpulmonary thermodilution device in place (PiCCO2, Pulsion Medical Systems, Feldkirchen, Germany), mechanical ventilation in the volume assist control mode with a Vt of 6 mL/kg of predicted body weight, adaption to the ventilator, and the decision taken by the clinicians in charge to perform volume expansion. This decision was made on the basis of clinical signs of inadequate tissue perfusion such as (1) systolic blood pressure < 90 mmHg (or a decrease > 50 mmHg in previously hypertensive patients), (2) urine output < 0.5 mL/kg/hour for at least 2 h, (3) tachycardia or (4) presence of skin mottling or increased capillary refill time. It also took into account the absence of excessive risk of fluid overload, as typically indicated by the level of central venous pressure, extravascular lung water and the cumulative fluid balance.

Patients were excluded if the PLR manoeuvre was contra-indicated (intracranial hypertension) or possibly unreliable (venous compression stocking, intra-abdominal hypertension [12]). Other exclusion criteria were spontaneously triggered cycles on the airway pressure waveform, cardiac arrhythmias, impossibility to obtain haemodynamic stability (defined by no change in the norepinephrine dose and no change in systolic arterial pressure < 10% within 5 min before the inclusion), poor echogenicity impeding the measurement of the IVC diameter and of the velocity time integral (VTI) in the left ventricular outflow tract.

### Echocardiographic measurements

IVC sonography was performed by a 4-year experienced intensivist (TT), who holds a university degree in echocardiography. With the 3.5-MHz cardiovascular ultrasound probe of a Philips CX50 device (Philips ultrasound system, Philips Healthcare, DA Best, The Netherlands), the IVC was examined in the subcostal window in longitudinal section in M-mode, 2 cm upstream of the origin of the hepatic veins.

The distensibility index of the IVC, which reflects the increase in its diameter on insufflation, was calculated as IVCDV = (maximum diameter on inspiration − minimum diameter on expiration)/(mean of maximum and minimum diameters).

The VTI was measured at end expiration in the left ventricular outflow tract on the apical five-chamber window. On the apical 4-chamber view, the left ventricular ejection fraction was calculated by the biplane method of disks summation (modified Simpson’s rule). The average of three consecutive cardiac cycles was used for all ultrasound measurements in case of sinus rhythm, and a representative cardiac cycle was chosen in case of atrial fibrillation [13]. Endocardial contours and VTI envelope were hand drawn.

### Haemodynamic measurements

All patients had a central venous catheter in the superior vena cava territory and a thermistor-tipped catheter inserted through the femoral artery. Transpulmonary thermodilution measurements were performed by injecting 15 mL cold normal saline (< 8 °C) through the central venous catheter. The average from three consecutive 15-mL injections was recorded at each time point [14] and was used to obtain CI, the global end-diastolic volume (marker of cardiac preload), the extravascular lung water and the cardiac function index (estimate of the left ventricular ejection fraction). Pulse contour analysis allowed the continuous and real-time calculation of CI after an initial calibration by thermodilution [15].

The intra-abdominal pressure was estimated from the bladder pressure. The transducer was zeroed and placed at the pubic symphysis [12].

### Study design

At baseline, all patients were in the 45° semi-recumbent position (Additional file 1: Supplementary Figure 2). A first set of thermodilution and echocardiographic measurements was performed, including CI (measured by thermodilution), PPV, SVV and IVCDV. Then, we performed a PLR test as previously described [8]. Pulse contour analysis-derived CI, PPV, stroke volume variation (SVV) and IVCDV were recorded at the maximal effect of PLR on CI, which occurs within 1 min [8]. A third set of measurements (CI (pulse contour analysis), PPV, SVV and IVCDV) was performed once patients were returned to the semi-recumbent position and a steady state was obtained again.

A “Vt challenge” was then performed by increasing Vt from 6 to 8 mL/kg of predicted body weight for 1 min [10]. A fourth set of measurements (CI (pulse contour analysis), PPV, SVV and IVCDV) was recorded once CI remained stable. Vt was then decreased back to 6 mL/kg of predicted body weight, and another set of measurements was performed after a new stable state was reached, including CI (thermodilution), PPV, SVV and IVCDV. Finally, in preload responsive patients, 500 mL of normal saline was infused over 10 min. In these patients, a last set of measurements was recorded after the end of fluid infusion (CI (thermodilution), PPV, SVV and IVCDV).

Except Vt, ventilatory settings and treatments were unchanged during the study period. The intrabdominal pressure and the central venous pressure were measured at each study step. The CI measured by transpulmonary thermodilution and pulse contour analysis was continuously recorded by the PiCCO Win 4.0 software (Pulsion Medical Systems). The intravascular, intra-abdominal and airway pressure signals were continuously recorded by using a data acquisition software (HEM 4.2, Notocord, Croissy-sur-Seine, France).

### Statistical analysis

Patients in whom PLR, performed at Vt = 6 mL/kg, induced an increase in CI (measured by pulse contour analysis) ≥ 10%, were defined as preload responders. Normality of data distribution was assessed visually. Variables were summarized as mean ± SD (if normally distributed), median and interquartile range (if non-normally distributed) or counts and percentages. Variables before and after fluid administration were compared by a paired Student *t* test (if normally distributed) or a Wilcoxon test (if non-normally distributed). Variables between preload responders and non-responders were compared using a two-sample Student t test (if normally distributed), a Mann–Whitney U test (if non-normally distributed), a Chi-square test or a Fisher exact test, as indicated.

Receiver operating characteristic (ROC) curves (with 95% confidence interval) were generated for quantifying the ability of the following variables to detect preload responsiveness: (1) IVCDV, PPV and SVV at baseline (Vt of 6 mL/Kg); (2) changes in IVCDV (ΔIVCDV_Vt_), in PPV (ΔPPV_Vt_) and in SVV (ΔSVV_Vt_) induced by the Vt challenge, expressed either as the change in absolute value (value during Vt challenge − value at baseline) or as the percent relative change from the baseline value ((value during Vt challenge − value at baseline)/value at baseline × 100); (3) changes in IVCDV (ΔIVCDV_PLR_), in PPV (ΔPPV_PLR_) and in SVV (ΔSVV_PLR_) induced by the PLR test, expressed either as the change in absolute value (value during PLR − value at baseline) or as the percent relative change from the baseline value ((value during PLR − value at baseline)/value at baseline × 100); the areas under ROC curves (AUROC) were compared by the Hanley–McNeil test. The best diagnostic threshold was determined as the one providing the best Youden index (sensitivity + specificity − 1). The echocardiographic measurements were performed offline without knowing the results of the PLR test, but the values of PPV were collected at the same time as CI, knowing its changes during the PLR test.

The least significant change of IVCDV was obtained from six successive measurements of IVCDV performed during haemodynamic stability at Vt = 6 mL/kg, by the same operator, removing the probe from the patient’s skin for each measurement, as previously described [13].

In order to demonstrate a significant difference between groups of ∆IVCDV_Vt_, assuming a precision of the IVC measurement of 12% [16, 17] with an α risk of 5% and a β risk of 20%, we planned to include 15 preload responders and 15 preload non-responders. Statistical analysis was performed with MedCalc 11.6.0 software (MedCalc, Mariakerke, Belgium).

## Results

### Patients

Forty-two patients were screened (Additional file 1: Supplementary Figure 3). Five were not included because of haemodynamic instability, and seven due to a poor ultrasound window. No patient was excluded for other reasons. Thirty patients were finally included and analysed. All patients with a positive PLR test received volume expansion. Their characteristics are detailed in Table 1. At the time of the study, propofol was administered in 28 (93%) patients and remifentanil in 25 (83%) patients. Neuromuscular blocking agents were used in six (20%) patients. The intra-observer variability of the measurement of IVCDV at baseline was 9 [3–18]%.

**Table 1:** Patient characteristics at baseline

	Preload responders (*n* = 15)	Preload non-responders (*n* = 15)	*p* value
Age (years)	63 ± 18	70 ± 10	0.37
Male gender (*n*, %)	12 (80.0)	11 (73.3)	0.32
SAPS2	52 ± 15	56 ± 14	0.29
Mortality (*n*, %)	7 (46.7)	6 (40.0)	1.00
Septic shock (*n*, %)	11 (73.3)	12 (80.0)	0.14
Cardiogenic shock (*n*, %)	2 (13.3)	2 (13.3)	0.55
Hypovolemic shock (*n*, %)	1 (7.2)	1 (7.2)	1.00
Vasoplegic shock (non-septic) (*n*, %)	1 (7.2)	0 (0.0)	0.12
CRRT (*n*, %)	3 (20.0)	4 (26.7)	0.18
ARDS (*n*, %)	6 (40.0)	5 (33.3)	0.47
Lactate (mmol/L)	1.8 ± 0.6	1.3 ± 0.6	0.88
PaO_2_/FiO_2_	228 ± 105	276 ± 105	0.55
PEEP (cmH_2_O)	10.7 ± 3.6	10.4 ± 3.0	0.63
*C*_rs_ (mL/cmH_2_O)	31 ± 12	32 ± 13	0.66
Acute cor pulmonale (*n*, %)	0 (0.0)	0 (0.0)	1.00
LVEF (%)	45 ± 9	49 ± 11	0.67
Patients receiving norepinephrine (*n*, %)	15 (100.0)	15 (100.0)	1.00
Dose of norepinephrine (µg/kg/min)	1.2 ± 0.6	0.6 ± 0.4	**0.02**

P values in bold: < 0.05

*ARDS* acute respiratory distress syndrome, *CRRT* continuous renal replacement therapy, *C*_*rs*_ compliance of the respiratory system, *LVEF* left ventricular ejection fraction, *PaO*_*2*_*/FiO*_*2*_ ratio of the arterial oxygen partial pressure over the oxygen inspired fraction, *PEEP* positive end-expiratory pressure, *SAPS* simplified acute physiologic score

### Changes in CI over study steps, characteristics of preload responders and non-responders

The PLR test (performed at Vt = 6 mL/kg) increased CI ≥ 10% in 15 preload responders. Patient characteristics are detailed in Table 1. Increasing Vt from 6 to 8 mL/kg decreased CI and VTI in preload responders, but not in preload non-responders (Table 2). In preload responders, fluid infusion increased CI ≥ 15% in all the patients.

**Table 2:** Haemodynamic variables at different study steps

		Baseline 1 (Vt = 6 mL/kg)	PLR (Vt = 6 mL/kg)	P PLR versus baseline 1	Baseline 2 (Vt = 6 mL/kg)	Vt challenge (Vt = 8 mL/kg)	P Vt challenge versus baseline 2	Baseline 3 (Vt = 6 mL/kg)	After VE (Vt = 6 mL/kg)	P After VE versus baseline 3
*HR (beats/min)*										
Preload responders		85 ± 18	77 ± 11	0.10	85 ± 17	88 ± 19	0.65	84 ± 17	90 ± 15	0.37
Preload non-responders		74 ± 18	74 ± 19	1.95	74 ± 18	75 ± 22	0.89			
*SAP (mmHg)*										
Preload responders		118 ± 17	124 ± 21	0.40	119 ± 17	116 ± 19	0.65	120 ± 17	129 ± 13	0.11
Preload non-responders		128 ± 16	126 ± 19	0.76	128 ± 16	130 ± 16	0.73			
*MAP (mmHg)*										
Preload responders		75 ± 12	79 ± 14	0.41	76 ± 12	75 ± 14	0.85	79 ± 13	83 ± 10	0.35
Preload non-responders		87 ± 12	86 ± 12	0.82	87 ± 12	85 ± 8	0.59			
*DAP (mmHg)*										
Preload responders		57 ± 10	59 ± 12	0.62	57 ± 11	59 ± 15	0.68	56 ± 11	58 ± 9	0.59
Preload non-responders		61 ± 10	60 ± 8	0.76	62 ± 9	69 ± 10	0.06			
*CVP (mmHg)*										
Preload responders		10 ± 3	11 ± 3	**< 0.01**	9 ± 5	10 ± 5	**< 0.01**	11 ± 3	12 ± 6	**< 0.01**
Preload non-responders		10 ± 2	12 ± 3	**< 0.01**	12 ± 4	11 ± 4	**< 0.01**			
* IAP (mmHg)*										
Preload responders		8 ± 3	7 ± 4	**0.04**	8 ± 3	8 ± 3	1.00	6 ± 2	6 ± 2	1.00
Preload non-responders		7 ± 4	6 ± 3	0.06	7 ± 3	7 ± 3	0.98			
*CI (L/min/m*^2^*)*										
Preload responders		3.0 ± 0.9	3.5 ± 1.3	**< 0.01**	3.0 ± 1.3	2.7 ± 1.1	**< 0.01**	2.8 ± 0.9	3.4 ± 1.2	**< 0.01**
Preload non-responders		3.0 ± 1.1	3.0 ± 0.9	0.78	3.0 ± 0.9	3.0 ± 0.8	0.39			
*Pplat (cmH*_2_*O)*										
Preload responders		22.2 ± 6.2	22.2 ± 6.2	1.00	22.2 ± 6.2	24.5 ± 5.4	**0.01**	22.3 ± 6.1	22.3 ± 6.1	1.00
Preload non-responders		22.9 ± 4.6	22.9 ± 4.6	1.00	22.9 ± 4.6	24.8 ± 5.2	**0.01**			
*PEEP (cmH*_2_*O)*										
Preload responders		10.7 ± 3.6	10.7 ± 3.6	1.00	10.7 ± 3.6	10.7 ± 3.6	1.00	10.7 ± 3.6	10.7 ± 3.6	1.00
Preload non-responders		10.4 ± 3.0	10.4 ± 3.0	1.00	10.4 ± 3.0	10.4 ± 3.0	1.00			
*Driving pressure (cmH*_2_*O)*										
Preload responders		11.5 ± 2.2	11.7 ± 2.3	0.90	11.5 ± 2.2	13.8 ± 1.9	**0.03**	11.5 ± 2.2	11.5 ± 2.2	1.00
Preload non-responders		12.3 ± 2.2	12.4 ± 2.2	0.95	12.3 ± 2.2	14.4 ± 2.4	**0.01**			
*PPV (%)*										
Preload responders		8 ± 3	5 ± 2	**< 0.01**	8 ± 3	13 ± 4	**< 0.01**	8 ± 2	4 ± 2	**< 0.01**
Preload non-responders		6 ± 3	6 ± 3	0.08	7 ± 3	6 ± 3	0.84			
*SVV (%)*										
Preload responders		8 ± 2	6 ± 2	**< 0.01**	8 ± 2	12 ± 3	**< 0.01**	8 ± 2	5 ± 2	**< 0.01**
Preload non-responders		6 ± 3	6 ± 3	0.43	6 ± 3	5 ± 2	0.31			
*IVCDV (%)*										
Preload responders		9 ± 3	7 ± 2	**0.01**	10 ± 2	15 ± 3	**< 0.01**	8 ± 2	6 ± 3	**0.04**
Preload non-responders		7 ± 3	6 ± 2	0.22	7 ± 3	6 ± 3	0.17			
*IVC max diam (cm)*										
Preload responders		2.0 ± 0.2	2.1 ± 0.2	**< 0.01**	1.9 ± 0.2	2.1 ± 0.2	0.26	1.9 ± 0.2	2.1 ± 0.3	**< 0.01**
Preload non-responders		2.0 ± 0.4	2.1 ± 0.4	**0.02**	2.3 ± 0.3	2.3 ± 0.3	0.84			
*VTI (cm)*										
Preload responders		20 ± 4	24 ± 6	**0.01**	21 ± 5	18 ± 6	**0.04**	20 ± 4	25 ± 5	**< 0.01**
Preload non-responders		21 ± 4	21 ± 4	0.89	21 ± 4	21 ± 4	0.89			
*EVLW (mL/Kg)*										
Preload responders		14 ± 7			14 ± 7			14 ± 6	13 ± 5	0.62
Preload non-responders		13 ± 4			13 ± 4					
*PVPI*										
Preload responders		2.6 ± 1.0			2.7 ± 1.0			2.7 ± 1.2	2.6 ± 1.0	0.81
Preload non-responders		2.5 ± 1.0			2.6 ± 1.0					
*GEDV (mL/m*^2^*)*										
Preload responders		725 ± 142			645 ± 100			695 ± 124	794 ± 135	**0.04**
Preload non-responders		821 ± 125			820 ± 178					
*CFI (*^−1^*)*										
Preload responders		4.1 ± 1.5			4.2 ± 1.7			4.2 ± 1.6	4.2 ± 1.6	0.85
Preload non-responders		4.5 ± 2.1			4.2 ± 1.6					

P values in bold: < 0.05

*CI* cardiac index, *CFI* cardiac function index, *CVP* central venous pressure, *DAP* diastolic arterial pressure, *EVLW* extravascular vascular lung water indexed for body weight, *GEDV* global end-diastolic volume indexed for body surface, *HR* heart rate, *IAP* intra-abdominal pressure, *IVC* inferior vena cava, *IVCDV*: inferior vena cava variation, *MAP* mean arterial pressure, *PEEP* positive end-expiratory pressure, *PLR* passive leg raising, *Pplat* plateau pressure, *PPV* pulse pressure variation, *PVPI* pulmonary vascular permeability index, *SAP* systolic arterial pressure, *SVV* stroke volume variation, *VE* volume expansion, *Vt* tidal volume, *VTI* velocity time integral

### Changes in IVCDV over study steps, detection of preload responsiveness through IVC indices

**Fig. 1:**
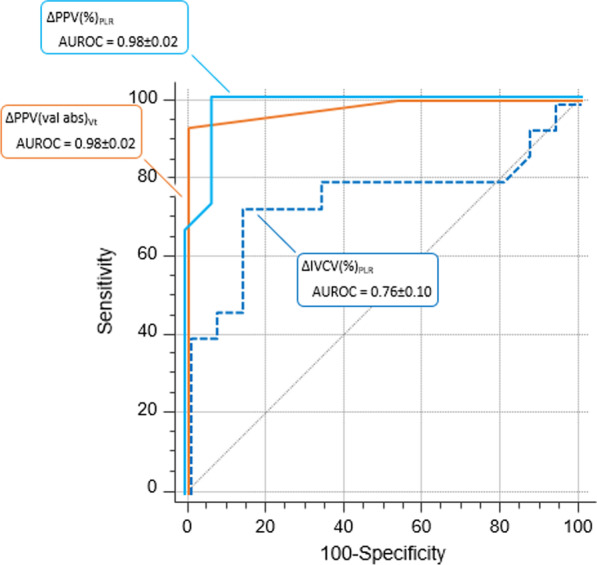
Receiver operating characteristic curves describing the ability to diagnose preload responsiveness of the changes in passive leg raising-induced changes of pulse pressure variation in percent (ΔPPV(%)_PLR_), passive leg raising-induced changes and of inferior vena cava variation in percent (ΔIVCDV(%)_PLR_), and of the tidal volume challenge-induced changes of pulse pressure variation in absolute value (ΔPPV(valabs)_Vt_). AUROC: area under the receiver operating characteristic curve (expressed as mean ± SD)

At baseline at Vt = 6 mL/kg, the end-expiratory IVC diameter as well as IVCDV were similar between preload responders and preload non-responders (Table 2). ΔIVCDV_PLR_ was larger in preload responders than in preload non-responders (Table 3, Additional file 1: Supplementary Figure 4). ΔIVCDV_PLR_ expressed in percent change from the baseline value reliably detected preload responsiveness, with a diagnostic threshold of − 24% (Table 4). ΔIVCDV_PLR_ was significantly correlated with the PLR-induced changes in CI. ΔIVCDV_PLR_ expressed in absolute change did not detect preload responsiveness (AUROC not different from 0.5) and was not correlated with the PLR-induced changes in CI (Table 4, Fig. 1).

**Table 3:** Indices of preload responsiveness at different study times in preload responders and non-responders

		Effects of PLR (Vt = 6 mL/kg)	Effects of the Vt challenge (Vt = 8 mL/kg)	Effects of VE (Vt = 6 mL/kg)
*ΔCI (% change)*				
Preload responders Preload non-responders		18 ± 6 4 ± 4	− 9 ± 8 2 ± 6	25 ± 9
P preload non-responders versus preload responders		**< 0.01**	**< 0.01**	
*ΔVTI (% change)*				
Preload responders Preload non-responders		16 ± 3 1 ± 1	− 10 ± 7 1 ± 7	+ 16 ± 8
P preload non-responders versus preload responders		**< 0.01**	**< 0.01**	
*ΔPPV (% change)*				
Preload responders Preload non-responders		− 38 ± 16 − 4 ± 8	64 ± 42 1 ± 28	− 50 ± 12
P preload non-responders versus preload responders		**< 0.01**	**< 0.01**	
*ΔPPV (absolute change)*				
Preload responders Preload non-responders		− 3 ± 1 0 ± 1	5 ± 2 0 ± 1	− 4 ± 2
P preload non-responders versus preload responders		**< 0.01**	**< 0.01**	
ΔSVV (% change)				
Preload responders Preload non-responders		− 24 ± 20 1 ± 15	44 ± 22 − 1 ± 3	− 40 ± 17
P preload non-responders versus preload responders		**< 0.01**	**< 0.01**	
ΔSVV (absolute change)				
Preload responders Preload non-responders		− 2 ± 2 0 ± 1	3 ± 2 − 1 ± 3	− 4 ± 2
P preload non-responders versus preload responders		**< 0.01**	**< 0.01**	
*ΔIVCDV (% change)*				
Preload responders Preload non-responders		− 26 ± 28 − 3 ± 20	91 ± 72 − 10 ± 52	− 25 ± 15
P preload non-responders versus preload responders		**0.02**	**< 0.01**	
*ΔIVCDV (absolute change)*				
Preload responders Preload non-responders		− 2 ± 3 − 1 ± 2	6 ± 4 − 1 ± 4	− 2 ± 4
P preload non-responders versus preload responders		0.51	**< 0.01**	

P values in bold: < 0.05

*ΔCI* percent changes in cardiac index, *ΔIVCDV* percent changes in the inferior vena cava diameter variation, *ΔPPV* percent changes in pulse pressure variation, *ΔSVV* percent changes in stroke volume variation, *ΔVTI* percent changes in velocity time integral

**Table 4:** Ability of different variables to detect preload responsiveness

	*r* versus PLR-induced changes in CI	*p* versus 0.5	AUROC	sd	*p* versus 0.50	Cut-off	Se	95% CI	Sp	95% CI	+ PV	95% CI	− PV	95% CI	+ LR	95% CI	− LR	95% CI
GEDV (mL/m^2^)			0.59	0.10	0.47													
CVP (mmHg)			0.56	0.11	0.57													
IVC max diameter (mm)			0.58	0.11	0.49													
PPV (%)			0.66	0.10	0.10													
SVV (%)			0.61	0.10	0.10													
IVCDV (%)			0.64	0.11	0.20													
ΔPPV_PLR_ (% change)	− **0.77**	**< 0.01**	**0.98**	**0.02**	**< 0.01**	**≤ **− **20%**	**100**	**78–100**	**93**	**61–100**	**94**	**69–99**	**100**		**15.0**	**2.3–99.6**	**0.0**	
ΔPPV_PLR_ (abs. change)	− **0.70**	**< 0.01**	**0.98**	**0.02**	**< 0.01**	**≤ **− **2 points**	**93**	**68–100**	**93**	**68–100**	**94**	**69–99**	**94**	**69–99**	**14.0**	**2.1–93.4**	**0.1**	**0.0–0.5**
ΔPPV_Vt_ (% change)	**0.53**	**< 0.01**	**0.94**	**0.04**	**< 0.01**	**> 20%**	**93**	**68–100**	**87**	**59–98**	**87**	**66–96**	**94**	**69–99**	**7.0**	**1.9–25.6**	**0.1**	**0.0–0.5**
ΔPPV_Vt_ (abs. change)	**0.71**	**< 0.01**	**0.98**	**0.02**	**< 0.01**	**> 1 point**	**93**	**68–100**	**100**	**78–100**	**100**		**94**	**69–99**			**0.1**	**0.0–0.5**
ΔSVV_PLR_ (% change)	− **0.66**	**< 0.01**	**0.90**	**0.07**	**< 0.01**	**≤ **− **19%**	**80**	**52–96**	**100**	**78–100**	**100**		**83**	**65–93**	**5.5**	**1.5–20.7**	**0.3**	**0.1–0.7**
ΔSVV_PLR_ (abs. change)	− **0.73**	**< 0.01**	**0.88**	**0.07**	**< 0.01**	**≤ **− **2 points**	**73**	**45–92**	**100**	**78–100**	**100**		**79**	**62–90**			**0.3**	**0.1–0.6**
ΔSVV_Vt_ (% change)	0.07	0.69	**0.82**	**0.08**	**< 0.01**	**> 20%**	**100**	**78–100**	**67**	**38–88**	**70**	**55–81**	**75**	**60–86**	**3.0**	**1.5–6.1**	**0.0**	
ΔSVV_Vt_ (abs. change)	0.21	0.26	**0.94**	**0.04**	**< 0.01**	**> 1 point**	**93**	**68–100**	**73**	**45–92**	**78**	**60–89**	**100**		**3.5**	**1.5–8.2**	**0.1**	**0.0–0.6**
ΔIVCDV_PLR_ (% change)	− **0.50**	**< 0.01**	**0.76**	**0.10**	**< 0.01**	**≤ **− **24%**	**73**	**45–92**	**87**	**85**	**83**	**59–95**	**77**	**58–89**	**5.5**	**1.5–20.7**	**0.3**	**0.1–0.7**
ΔIVCDV_PLR_ (abs. change)	− 0.30	0.09	0.56	0.11	0.60													
ΔIVCDV_Vt_ (% change)	**0.52**	**< 0.01**	**0.88**	**0.06**	**< 0.01**	**> 4%**	**100**	**78–100**	**67**	**38–88**	**75**	**60–86**	**100**		**3**	**1.5–6.1**	**0.0**	
ΔIVCDV_Vt_ (abs. change)	**0.57**	**< 0.01**	**0.92**	**0.05**	**< 0.01**	**> 1 point**	**100**	**78–100**	**60**	**32–84**	**71**	**57–83**	**100**		**2.5**	**1.3–4.6**	**0.0**	

*AUROC* area under the receiver operating characteristic curve, *CI* confidence interval, *GEDV* global end-diastolic volume indexed for body surface, *IVC* inferior vena cava, *IVCDV* respiratory variation of the diameter of the inferior vena cava, *PPV* pulse pressure variation, *Se* sensitivity, *sd* standard deviation, *Sp* specificity, *SVV* stroke volume variation,* + PV* positive predictive value, − *PV* negative predictive value, + *LR* positive likelihood ratio, − *LR* negative likelihood ratio, *ΔPPV*_*PLR*_ changes in pulse pressure variation induced by passive leg raising, *ΔSVV*_*PLR*_ changes in stroke volume variation induced by passive leg raising, *ΔIVCDV*_*PLR*_ changes in the respiratory variation of the diameter of the inferior vena cava induced by a tidal volume challenge, *ΔPPV*_*Vt*_ changes in pulse pressure variation induced by a tidal volume challenge, *ΔSVV*_*Vt*_ changes in stroke volume variation induced by a tidal volume challenge, *ΔIVCDV*_*Vt*_ changes in the respiratory variation of the diameter of the inferior vena cava induced by a tidal volume challenge

ΔIVCDV_Vt_ was larger in preload responders than in preload non-responders (Table 3, Additional file 1: Supplementary Figure 4). ΔIVCDV_Vt_ expressed in percent change from the baseline value reliably detected preload responsiveness, with a diagnostic threshold of 4% (Table 4). This was also the case for ΔIVCDV_Vt_ expressed in absolute change (Table 4, Fig. 1). Both indices were correlated with the PLR-induced changes in CI (Table 4).

### Changes in PPV and SVV over study steps, detection of preload responsiveness through PPV/SVV indices

ΔPPV_PLR_ was larger in preload responders than in preload non-responders (Table 3, Additional file 1: Supplementary Figure 4). The PLR-induced changes in PPV reliably detected preload responsiveness, either expressed in percent change from baseline (Table 4) or in absolute change (Table 4, Fig. 1). Both indices were correlated with the PLR-induced changes in CI (Table 4). Similar results were observed for ΔSVV_PLR_ (Table 4).

ΔPPV_Vt_ was larger in preload responders than in preload non-responders (Table 3, Additional file 1: Supplementary Figure 4). The Vt challenge-induced changes in PPV reliably detected preload responsiveness, either expressed in percent change from baseline (Table 4) or in absolute change (Table 4, Fig. 1). Both indices were correlated with the PLR-induced changes in CI (Table 4).

ΔSVV_Vt_ was larger in preload responders than in preload non-responders (Table 3, Additional file 1: Supplementary Figure 4). The Vt challenge-induced changes in SVV reliably detected preload responsiveness, either expressed in percent change from baseline (Table 4) or in absolute change (Table 4, Fig. 1). However, both indices were not correlated with the PLR-induced changes in CI (Table 4).

### Comparisons of ROC curves

The AUROC for ΔIVCDV_PLR_ expressed in absolute value was significantly lower than the AUROC of any other index (ΔIVCDV_Vt_, ΔPPV_Vt_, ΔSVV_Vt_, ΔPPV_PLR_ and ΔSVV_PLR_ expressed in percent change or in absolute value and ΔIVCDV_PLR_ expressed in percent change). There was no significant difference of AUROC between all other indices.

## Discussion

In this study performed in critically ill patients, we show that the PLR-induced decrease in IVCDV has a reliable diagnostic value but only expressed in percent change and that the increase in IVCDV during a Vt challenge may detect preload responsiveness, but with a diagnostic threshold far lower than the least significant change of IVCDV. Along with a previous study [18], we also suggest the PLR-induced decrease in PPV detects preload responsiveness, we suggest that the variations in IVC diameter with mechanical ventilation are poor markers of preload responsiveness in case of Vt = 6 mL/kg, and we show that the increase in PPV during a Vt challenge detects preload responsiveness.

Several tests are today available for detecting preload responsiveness and predicting the response of cardiac output to fluid infusion [3]. Nevertheless, they differ regarding their conditions of use and the monitoring devices that are required to assess their effects. PPV and SVV are reliable, but their reliability is severely decreased in case of spontaneous breathing, cardiac arrythmias, low lung compliance and Vt < 8 mL/kg [1]. The PLR test has a similar reliability [19, 20], but its main drawback is that its effects cannot be assessed simply on systolic or pulse arterial pressure [8]. The present study describes how PPV and SVV could be used to assess preload responsiveness in case of low Vt < 8 mL/kg, and how the effects of the PLR test can be assessed without measuring cardiac output directly.

First, our findings suggest the IVCDV was not a reliable indicator of preload responsiveness in case of Vt = 6 mL/kg, as it has been already shown by a previous study [6] (Additional file 1: Supplementary Table 1). The changes in IVC dimensions under mechanical ventilation are due to the cyclic changes in its transmural pressure created by the changes in central venous pressure and likely in intra-abdominal pressure. Then it is not surprising that a low Vt, inducing lower changes in intrathoracic and transmural pressures, is responsible for a lower diagnostic ability compared to Vt ≥ 8 mL/kg. Nevertheless, it is important to emphasize that the reliability of IVCDV for detecting preload responsiveness has been found to be poor or moderate by many studies and meta-analyses, even in studies including patients with Vt ≥ 8 mL/kg [21, 22]. Along with these studies, the present one shows that IVCDV is likely the dynamic index of fluid responsiveness with the poorest diagnostic value.

Second, we found that the PLR-induced decrease in PPV reliably detected preload responsiveness, whatever the way it was calculated. This was the case when expressed either in absolute or in relative change, and ΔPPV_PLR_ was the index with the highest correlation with the degree of preload responsiveness, as assessed by the PLR-induced changes in CI. ΔSVV_PLR_ provided similar results, though the correlation with preload responsiveness intensity was a bit lower. This result might be of clinical importance. Indeed, PLR, the main alternative to PPV and SVV in case of Vt < 8 mL/kg, requires a direct measurement of cardiac output [8] and many studies attempted to find cardiac output surrogates that may be used for this purpose. Provided that the patient is equipped with an arterial catheter, PPV is readily available and assessing the effects of PLR on it might be very easy. In this regard, this result should be compared to the assessment of the PLR test through the perfusion index of plethysmography [23] or its respiratory variation [24].

Third, the PLR-induced decrease in IVCDV detected preload responsiveness but only when expressed in percent change. Even in this way, the predictive ability was not excellent: the AUROC was 0.76 ± 0.10, tending to be lower than for the PLR-induced changes in PPV. The correlation with the PLR-induced changes in CI was only − 0.50. When IVCDV changes were expressed in absolute value, it changes during PLR were no more able to detect preload responsiveness. This is not surprising, as IVCDV is itself a poorer index of preload responsiveness than PPV. Then, its relative changes during preload manipulations must be poorer than the changes in PPV. Also, moving the patient to the PLR position undoubtedly introduces a difficulty in the measurement of IVCDV, which can only contribute to hamper its diagnostic value.

Fourth, we suggest that the Vt challenge is a reliable means to test preload responsiveness in case of low Vt, as it has been already shown [10, 25]. The diagnostic threshold expressed in absolute value (1%) was lower than already observed (3.5%) [10], and like the one reported by Messina* et al*. [25]. This point is very important, because a 1-point change is very low regarding the mean of PPV value. This may induce diagnostic mistakes, especially in patients in whom PPV is unstable. The effects of the Vt challenge on SVV were worse, which is not surprising as SVV results from an estimation of stroke volume from arterial pulse pressure [15]. The AUROC tended to be smaller than that for ΔPPV_Vt_, and the correlation between the PLR-induced changes in CI and ΔSVV_Vt_ was not significant.

In theory, preload responsiveness observed at Vt = 8 mL/kg should not imply that it also exists at Vt = 6 mL/kg, as increasing Vt has changed the degree of preload responsiveness. In theory, there might be some false positives to the Vt challenge when assessing preload responsiveness. Nevertheless, our results suggest that this is not a significant limitation, likely because the change in cardiac preload is not of enough amplitude for transforming a preload responsive patient at Vt = 8 mL/kg in a preload non-responsive patient at Vt = 6 mL/kg. We observed no false positives when using ΔPPV_Vt_ to assess preload responsiveness.

Of note, the Vt challenge induced very large increases in PPV, SVV and IVCDV, despite the respiratory driving pressure only slightly increased. The Vt challenge induced changes in PPV, SVV and IVCDV were much larger than these induced by PLR, although PLR increases cardiac preload to a larger extent. This might be explained by the fact that PLR increases cardiac preload, moving the equilibrium point rightward on the cardiac function curve, where it is flatter. By contrast, because it decreases cardiac preload, the Vt challenge moves the equilibrium point leftward, where the curve is steeper. This makes changes the respiratory changes in stroke volume (and PPV) larger (see Additional file 1: Figure 1).

Fifth, the results regarding the changes in IVCDV during a Vt challenge were disappointing. The AUROC was significantly different from 0.5, for absolute as for relative changes, but the diagnostic threshold was much lower than the least significant change of IVCDV we calculated. Also, the correlation between ΔIVCDV_Vt_ and the PLR-induced changes in CI was weak. This is a disappointing result, because it means that the Vt challenge can be performed only if an arterial line is present.

The first limitation of the study is that we assessed preload responsiveness through the effects of a PLR test and not through a fluid challenge. This is explained by ethical reasons, as it would be today unacceptable to plan fluid infusion in preload unresponsive patients only for research purposes. Nevertheless, one must admit that the reliability of the PLR test has been well established by a number of previous studies [19, 20]. Second, we did not investigate the superior vena cava collapsibility, which is an equivalent of IVCDV [26]. Third, the dose of norepinephrine was higher in preload responders than in preload non-responders. This may have impaired the comparability between groups in terms of IVC variability, because norepinephrine decreases the IVC compliance, and in terms of PLR-induced increases in cardiac preload, because norepinephrine may decrease the volume of venous blood mobilized during the PLR test. Fourth, we did not assess the “grey zone” of the tests we investigated, which may avoid binary decisions when using such tests [27]. Finally, we did not include in our analysis some other interesting tests predicting fluid responsiveness, such as for instance the recruitment manoeuvres.

## Conclusion

The present study suggests that IVCDV is not a reliable indicator of preload responsiveness in patients with Vt at 6 mL/kg. It describes how the changes in IVCDV, like the changes in PPV, induced by a PLR test and by a transient increase in Vt from 6 to 8 mL/kg detect preload responsiveness assessed at 6 mL/kg.

## Supplementary Information

**Additional file 1.** Supplemental Figure 1: Principle of the hypothesis; Supplemental Figure 2: Study Protocol; Supplemental Figure 3: Flow Chart; Supplementary figure 4; Supplemental Table 1: Characteristics of Studies.

## Data Availability

Individual, de-identified participant data are available from the corresponding author on reasonable request.
