# Supplementary material for: Do changes in pulse pressure variation and inferior vena cava distensibility during passive leg raising and tidal volume challenge detect preload responsiveness in case of low tidal volume ventilation?
Source: Crit Care. 2021 Mar 18;25:110. doi: 10.1186/s13054-021-03515-7 (PMC7972024; doi:10.1186/s13054-021-03515-7)
Supplement: Supplementary file 1 — Additional file 1. Supplemental Figure 1: Principle of the hypothesis; Supplemental Figure 2: Study Protocol; Supplemental Figure 3: Flow Chart; Supplementary figure 4; Supplemental Table 1: Characteristics of Studies. [file 13054_2021_3515_MOESM1_ESM.docx]

**Do changes in pulse pressure variation and inferior vena cava distensibility during passive leg raising and tidal volume challenge detect preload responsiveness in case of low tidal volume ventilation?**

Temistocle TACCHERI, MD^1,2^; Francesco GAVELLI, MD^1,2^; Jean-Louis TEBOUL, MD, PhD^1,2^; Rui SHI MD^1,2^; Xavier MONNET, MD, PhD^1,2^.

1. Hôpitaux universitaires Paris-Sud, Hôpital de Bicêtre, APHP, Service de médecine intensive - réanimation, 78, rue du Général Leclerc, Le Kremlin-Bicêtre, F-94270 France

2. Inserm UMR S_999, Univ Paris-Sud, 78, rue du Général Leclerc, Le Kremlin-Bicêtre, F-94270 France

Supplemental digital content

Supplementary figure 1: Principle of the hypothesis

Supplementary figure 2: Study protocol

Supplementary figure 3: Flow chart

Supplementary figure 4

*Upper panel*: PLR-induced percent changes in pulse pressure variation during a passive leg raising test (ΔPPV_PLR_) (expressed in percent changes relative to baseline) and a tidal volume challenge (ΔPPV_Vt_) (expressed in absolute changes) in preload responders and non-responders.

*Bottom panel*: PLR-induced percent changes in inferior vena cava variation during a passive leg raising test (ΔIVCV_PLR_) (expressed in percent changes relative to baseline) and a tidal volume challenge (ΔIVCV_Vt_) (expressed in absolute changes) in preload responders and non-responders.

* p<0.05 preload responders *vs.* preload non-responders.
